# Sagliker Syndrome: A Case Report of Facial Deformities and Renal Osteodystrophy Secondary to Hyperparathyroidism in End-Stage Renal Disease

**DOI:** 10.7759/cureus.64399

**Published:** 2024-07-12

**Authors:** José Manuel García Romero, Pedro Hugo Guerrero Morales, Maria Fernanda Rico Razo, José Macario Córdova Argueta, Erick Olaya Niebla

**Affiliations:** 1 Transplant and Donation Department, Regional General Hospital 1 of the Mexican Social Security Institute, Querétaro, MEX

**Keywords:** renal osteodystrophy, case report, secondary hyperparathyroidism, sagliker syndrome, end-stage renal disease

## Abstract

Sagliker syndrome (SS) is a rare but distinctive form of renal osteodystrophy associated with poorly managed secondary hyperparathyroidism (SHPT) in patients with chronic kidney disease (CKD). We present a case of a 28-year-old male with end-stage CKD on hemodialysis for 10 years, who exhibited progressive facial deformities and maxillofacial bone pain. Physical examination revealed bilateral expansion of the maxillary and mandibular bones and facial asymmetry. Radiological findings included diffuse bone thickening and multilocular cysts in the maxillofacial bones, while laboratory tests showed decreased levels of calcium and elevated parathyroid hormone, confirming SHPT. Despite multidisciplinary management involving nephrology, endocrinology, and maxillofacial surgery, the patient's condition deteriorated and he manifested community-acquired pneumonia leading to cardiopulmonary arrest and death. This case underscores the challenges in managing severe HPT in CKD and emphasizes the importance of early assessment and comprehensive multidisciplinary care to prevent irreversible complications.

## Introduction

Sagliker syndrome (SS) is a rare manifestation of renal osteodystrophy associated with uncontrolled secondary hyperparathyroidism (SHPT) in patients with chronic kidney disease (CKD). First described by Sagliker et al. in 2004, it is estimated to affect approximately 0.5% of hemodialysis patients, predominantly in young women aged 18-39 years. The prevalence of renal osteodystrophy in patients with CKD varies widely depending on the stage of the disease and the specific population studied. It is estimated that as many as 90% of patients with advanced CKD may experience some form of renal osteodystrophy. Poor management exacerbates this condition, leading to an imbalance in calcium and phosphorus levels. This imbalance can stimulate the development of SHPT and contribute to advanced conditions such as SS [1]. Genetic predisposition is implicated in the development of renal osteodystrophy, a multifactorial complication often associated with elevated parathyroid function in patients with CKD, leading to bone morphology alterations and systemic effects.

Several mechanisms are involved in the imbalance of calcium levels in CKD. Firstly, the loss of nephrons reduces renal functional capacity, decreasing the kidney's ability to reabsorb calcium, thereby increasing urinary losses and serum phosphorus accumulation. Additionally, declining renal function lowers levels of calcitriol, crucial for proper vitamin D function in calcium absorption in the intestines. Together, these factors perpetuate chronic low calcium levels, continuously stimulating parathyroid hormone activation, leading to ongoing bone resorption, and contributing to renal osteodystrophy [2,3]. The association level depends mainly on the intensity, the severity, and the duration of the disease.

Many patients, particularly those who develop end-stage renal disease at a young age, often face a high risk without being fully aware of the progression, as symptoms tend to accumulate gradually and subtly. A significant number of cases are reported from developing countries and involve young patients who lack access to optimal CKD therapies. Interestingly, these reports do not consistently highlight genetic predispositions as contributing factors [4]. Inadequately treated SHPT has been identified as a significant risk factor for the development of SS. This condition is characterized by severe facial deformities and may be associated with the formation of brown tumors [5,6]. The phenotypical features of these patients include irregular malformations of the maxillary, mandibular, and nasal bones, resulting in a disfigured appearance often referred to as "squirrel face." Additionally, about half of the fatal cases of SS are linked to cardiovascular and cerebrovascular complications stemming from excessive bone resorption and abnormal calcification of blood vessels [7].

## Case presentation

**Figure 1:**
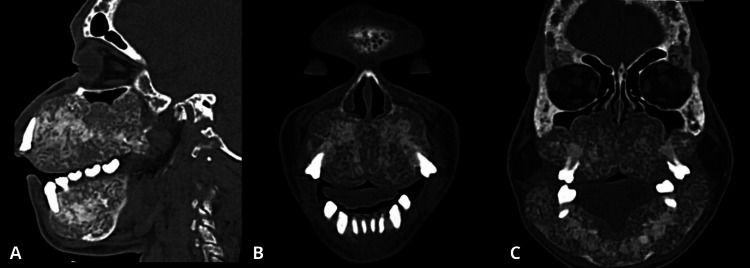
Head CT A: Sagittal view of maxilla displaying overgrowth of maxillary and mandibular bones with extensive lesions with a lithic appearance. B: Coronal view of the face at the nasal septum level showing diffuse enlargement of the hard palate with superimposed bony permeative change and spaced teeth. C: Coronal section at the level of the orbital cavity showing overgrowth of the maxilla and mandible bones profoundly affected by diffuse bone abnormalities CT: computed tomography

A 28-year-old male with end-stage chronic kidney disease, undergoing hemodialysis for a decade, presented with progressive facial deformities and maxillofacial bone pain. Physical examination revealed bilateral expansion of the maxillary and mandibular bones, facial asymmetry, widening of nasal fossae, dental spacing, and flattening of nasal cartilage. Palpation elicited tenderness in the affected areas. There were no signs of bone fracture or secondary neurological compromise. Radiological studies revealed distinctive features including increased bone resorption, multilocular bone cysts, osteolytic changes, and diffuse bone thickening in the mandibular and maxillary regions, resulting in facial asymmetry, deformity, and enlargement of the dental arch (Figures 1A-1C).

**Figure 2:**
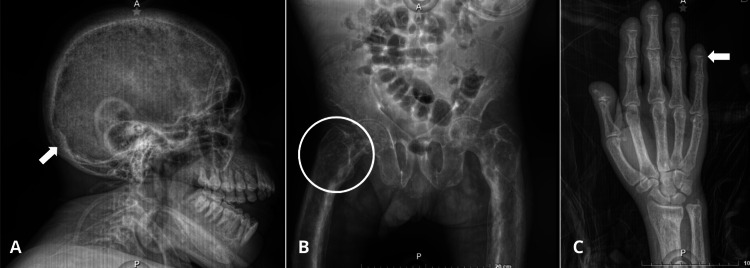
X-rays of the skull, hip, and hand A: Lateral skull X-ray showing calvarial thickening (white arrow) and multiple lytic irregularly circumscribed lesions as well as malocclusion of the upper and lower jaws. B: Anteroposterior hip X-ray showing long bone deformities, areas of osteitis fibrosa cystica (white circle), and O-shaped legs conditioning short stature. C: Anteroposterior X-ray of the hand showing fingertip deformity (white arrow)

**Figure 3:**
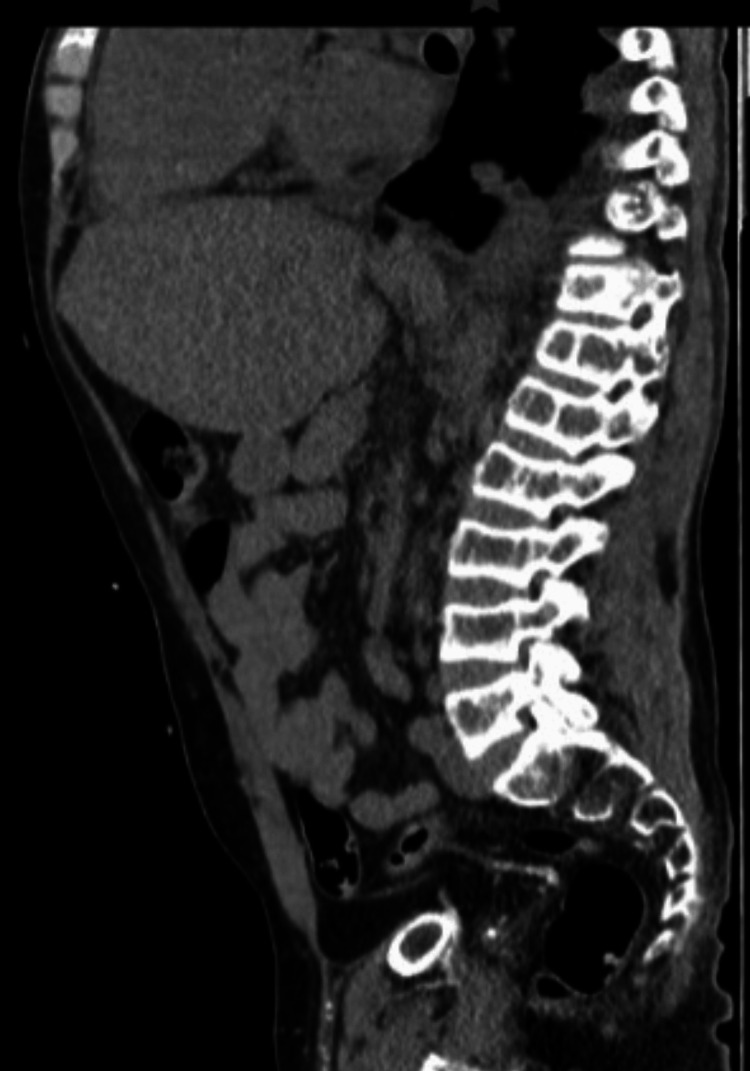
Sagittal non-contrast axial CT Sagittal section showing osteolysis, osteoporosis, and sclerosis in the lumbar vertebrae CT: computed tomography

Additionally, the findings indicated systemic involvement extending to the skull, hips, limbs, and spine, highlighting the extensive systemic impact of the condition (Figures 2A-2C, 3).

**Figure 4:**
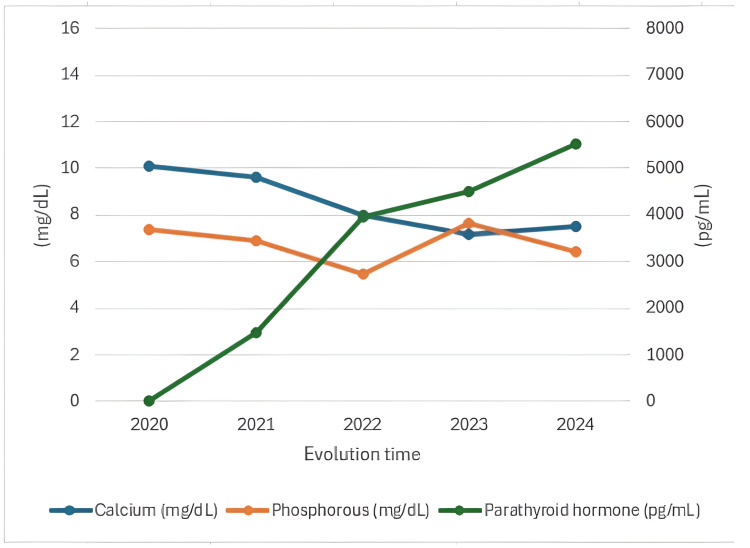
Patterns of parathyroid hormone and calcium-phosphorus ions Dual-axis graph illustrating the relationship between calcium and phosphorus ions alongside parathyroid hormone behavior, demonstrating a progressive decline in calcium levels accompanied by phosphorus levels exceeding the reference values, and a steady increase in parathyroid hormone levels over the years

As shown in Figure 4, which depicts laboratory values since 2020, a typical pattern of secondary hyperparathyroidism was evident, characterized by declining calcium levels as the disease advances, in contrast to increasing phosphorus and parathyroid hormone levels.

Concurrently, there was minimal improvement in renal function, as evidenced by elevated creatinine levels, alongside a progressive decline in hemoglobin levels over time (Table 1).

**Table 1:** Historical laboratory measurements Laboratory results show characteristics of secondary hyperparathyroidism and end-stage renal failure, including low hemoglobin levels, elevated azotemia, as well as increased levels of alkaline phosphatase and parathyroid hormone

	2020	2021	2022	2023	2024	Reference range
Parathyroid hormone (pg/mL)	NA	1469	3973.5	4517.7	5517.7	(15.0-68.3)
Calcium (mg/dL)	10.06	9.6	8	7.2	7.5	(8.30-10.60)
Phosphorus (mg/dL)	7.4	6.87	5.46	7.65	6.45	(2.4-5.1)
Alkaline phosphatase (U/L)	1334	NA	1420	955	1131	(45.00-129.00)
Hemoglobin (g/dl)	7.33	8.1	7.84	6.11	6.25	(11.0-18.0)
Urea (mg/dL)	141.66	143	92	96.12	86.5	(20.00-43.00)
Creatinine (mg/dL)	11.63	10.55	5.9	7.1	8	(0.50-1.10)

Initial management included optimizing dialysis therapy and adjusting phosphate-binding agents. The patient was then referred to a multidisciplinary team comprising nephrology, endocrinology, and maxillofacial surgery for collaborative management and long-term follow-up. Regular evaluations were planned to monitor the progression of bone deformities and treatment response. However, on April 24, 2024, the patient was admitted due to a 10-day history marked by dyspnea, nausea, desaturation, fever, and purulent sputum, leading to the initiation of broad-spectrum antibiotic therapy. Despite intensive efforts, clinical improvement was not achieved, and the patient experienced cardiopulmonary arrest due to septic shock and passed away on April 25, 2024.

## Discussion

While most theories propose a multifactorial origin for SS, its exact remains uncertain [8]. Nearly half of CKD patients, when not receiving appropriate treatment, may develop SHPT. Elevated levels of parathyroid hormone predispose patients to hyperphosphatemia, and concurrent deficiencies in active vitamin D, anemia, and hypocalcemia contribute to the hyperplasia and hypertrophy of the parathyroid gland. Elevated alkaline phosphatase and intact parathyroid hormone levels are also commonly observed [9,10]. Missense mutations in the exons of the GNAS1 gene on chromosome 20 have been reported in nearly half of the patients, correlating with severe hyperparathyroidism and likely contributing to abnormal bone growth. Studies have highlighted mutations in genes such as GNAS1, FGF23, and FGFR3, which are associated with bone dysplasia or hereditary dystrophies in CKD [7,11-13].

The overgrowth of craniomaxillofacial bones is primarily attributed to the activation of intramembranous ossification in this area [13]. Oropharyngeal manifestations include severe dental abnormalities with irregular positioning, benign tumors, and hyperplasia of soft tissues in the oral cavity, along with maxillary malocclusion. Other characteristic features include short stature, chest deformities, retraction of the nasal bridge, and deformities of the fingers, knees, and scapulae. Hearing impairment and severe neuropsychiatric disorders like depression are also commonly observed. Our patient initially presented with progressive bone pain followed by deformity of the mandibular and maxillary bones, which subsequently led to facial asymmetry, tooth spacing, and the growth of connective soft tissue around the jaw. Additionally, structural malformations at the femur and hip levels contributed to the worsening of short stature. Furthermore, SHPT, particularly when present from an early age, is associated with a range of musculoskeletal, immune, and cardiovascular disorders [14-16].

Although renal transplantation can slow the progression of musculoskeletal changes in SS, established deformities are generally irreversible and significantly impact patients' quality of life. The optimal management of SHPT remains contentious. Total parathyroidectomy is the definitive method to halt disease progression, though it does not reverse skeletal changes. Despite this limitation, it is preferred to prevent the persistence and recurrence of SHPT. Unfortunately, this treatment carries the risk of inducing refractory hypoparathyroidism [17]. Individualized treatment plans are crucial to determine the most suitable approach for each patient's specific circumstances.

SS, characterized by renal osteodystrophy, presents a severe manifestation of SHPT in CKD patients, profoundly affecting the quality of life and facial function. The notable bone involvement and long bone deformities associated with this syndrome have significant implications for morbidity, mortality, and mental health. Early diagnosis and comprehensive multidisciplinary management are essential to mitigate serious complications such as pathological fractures and to enhance long-term outcomes [18,19].

## Conclusions

This case report highlights the critical importance of considering SS in patients with CKD experiencing progressive facial bone changes. Timely and appropriate management of calcium and phosphate levels plays a crucial role in preventing SHPT and the subsequent development of facial renal osteodystrophy. If left untreated, this rare condition can profoundly impact patients both physically and psychologically, significantly reducing their quality of life. Hence, proactive monitoring and a comprehensive approach integrating medical and surgical interventions are essential to mitigate these complications and improve patient outcomes. This includes not only maintaining adequate control of calcium and phosphorus levels but also closely monitoring physical changes in patients to promptly initiate intensive measures aimed at halting bone deterioration, which can significantly impact both function and psychosocial well-being.
